# West Nile Virus in People with HIV: Immune Vulnerability, Neuroinvasive Disease, and Diagnostic Challenges

**DOI:** 10.3390/tropicalmed11090250

**Published:** 2026-09-03

**Authors:** Paolo Fusco, Francesco De Maria, Alessandro Russo

**Affiliations:** 1 Infectious and Tropical Diseases Unit, “Renato Dulbecco” Teaching Hospital of Catanzaro, Viale Europa, 88100 Catanzaro, Italy; paolofusco89@gmail.com; 2 Department of Medical and Surgical Sciences, “Magna Graecia” University of Catanzaro, Campus Universitario “Salvatore Venuta”, Viale Europa, 88100 Catanzaro, Italy; francescodemaria16@gmail.com

**Keywords:** West Nile virus, people with HIV, neuroinvasive disease, immune dysfunction, advanced HIV disease, diagnostic testing, molecular diagnosis, seroprevalence, prevention

## Abstract

West Nile virus (WNV) is an expanding mosquito-borne orthoflavivirus and an important cause of arboviral neuroinvasive disease. Whether HIV infection modifies WNV susceptibility, severity, or diagnostic performance remains uncertain. This narrative review was supported by structured PubMed/MEDLINE and Scopus searches updated to 22 August 2026 and a supplementary targeted citation search, identifying 23 peer-reviewed reports providing direct clinical or seroepidemiological evidence on WNV in people with HIV (PWH). The evidence remains heterogeneous, but recent data clarify several conclusions. A Northern Italian sero-repository study of 2843 PWH found neutralization-confirmed WNV antibodies in 3.0%, with no neuroinvasive disease and no association with HIV-related immunological variables. Comparative US cohorts likewise do not demonstrate that HIV status alone confers a uniform excess risk of WNV neuroinvasive disease. Conversely, individual reports of advanced HIV disease describe severe neuroinvasive presentations with delayed or absent WNV-specific antibody responses and molecular confirmation. HIV status alone should therefore not be considered a uniform WNV risk marker; the individual immune phenotype may be more clinically informative. In PWH with profound immunosuppression, negative early serology should not prematurely exclude WNV, and molecular testing should be considered when clinical suspicion remains high. Prevention, seasonal vigilance, and prospective HIV-specific studies remain priorities.

## 1. Introduction

West Nile virus (WNV) is an enveloped, positive-sense single-stranded RNA virus in the family Flaviviridae. Following recent taxonomic revision, the former genus Flavivirus has been renamed Orthoflavivirus, while established virus names such as West Nile virus remain unchanged [1]. WNV is maintained through an enzootic cycle involving birds as amplifying hosts and Culex mosquitoes as the principal vectors; humans and horses are generally incidental dead-end hosts [2,3].

WNV has become a persistent arboviral threat in North America and an increasingly important seasonal infection across Europe, the Mediterranean basin, and neighboring regions [4]. During the 2026 European transmission season, WHO/Europe highlighted an early acceleration of activity: by 22 July, Greece had reported 21 confirmed human cases, including 20 with neuroinvasive disease and 13 still hospitalized, while Italy had reported 21 cases across 17 areas by 15 July; locally acquired cases were also reported in North Macedonia, Romania, and Spain [5]. Transmission intensified thereafter. By 13 August, ECDC had recorded 429 locally acquired human WNV infections across nine European countries—Italy (224), Greece (105), Spain (42), North Macedonia (30), Romania (18), France (6), Serbia (2), Germany (1), and Kosovo (1) [6]. These provisional figures illustrate the rapid seasonal and geographical expansion of WNV activity and reinforce the importance of surveillance, vector control, and clinical awareness.

Most human WNV infections are asymptomatic. Among symptomatic individuals, the usual presentation is a self-limited febrile illness, whereas fewer than 1% develop West Nile neuroinvasive disease (WNND), including meningitis, encephalitis, acute flaccid myelitis/paralysis, or mixed neurological syndromes [7,8]. Advanced age, selected chronic comorbidities, hematological malignancy, transplantation, and immunosuppressive therapies are recognized correlates of severe disease [7,9,10]. However, the degree to which HIV infection contributes independently to this risk remains uncertain.

PWH have rarely been examined as a distinct population in the WNV literature. This knowledge gap is clinically relevant because HIV infection encompasses markedly heterogeneous immune phenotypes, ranging from durable virological suppression with substantial immune recovery to advanced HIV disease with profound CD4+ T-cell depletion, B-cell dysfunction, chronic immune activation, immunosenescence, and additional immunosuppressive exposures [11,12,13]. These abnormalities overlap biologically with mechanisms involved in WNV control, but mechanistic plausibility should not be equated with evidence that HIV status itself increases WNV susceptibility or severity.

Late HIV diagnosis further strengthens the clinical relevance of this intersection. Late presentation has traditionally been defined as presentation for HIV care with a CD4+ T-cell count below 350 cells/µL or an AIDS-defining event, whereas presentation with advanced HIV disease is defined by a CD4+ T-cell count below 200 cells/µL or an AIDS-defining condition [14,15,16]. Late diagnosis remains common in Europe and other settings [17,18,19]. Consequently, WNV may occasionally be encountered at the same time as newly diagnosed, profoundly immunocompromised HIV infection, creating a complex neurological differential diagnosis and potentially altering the performance of antibody-based testing.

Against this background, the present review critically synthesizes the direct clinical and seroepidemiological evidence on WNV infection in PWH and interprets it in the context of WNV immunopathogenesis, HIV-associated immune dysfunction, current diagnostic guidance, and prevention. The novelty of this review lies in explicitly separating direct WNV–HIV evidence from contextual mechanistic inference, incorporating newly available HIV-specific seroepidemiological and comparative data, and proposing a phenotype-based framework for clinical interpretation. Particular attention is given to geographical burden, advanced HIV disease, seronegative neuroinvasive presentations, molecular diagnosis, One Health prevention, and future antibody-based countermeasures.

## 2. Methods and Scope of the Review

This manuscript was developed as a narrative review supported by a structured literature search. The review was not designed as a formal systematic review or meta-analysis because the direct WNV–HIV evidence base is small and methodologically heterogeneous, comprising case reports and clinical observations, seroepidemiological studies, clinicopathological and syndrome-based cohorts, and retrospective comparative analyses. To improve transparency during revision, a PRISMA-style selection process was applied specifically to the direct clinical and seroepidemiological evidence component [20].

PubMed/MEDLINE and Scopus were searched from database inception to 22 August 2026. The PubMed/MEDLINE strategy combined “West Nile Virus” or “West Nile Fever” Medical Subject Headings and the terms “West Nile virus”, “West Nile fever”, “West Nile neuroinvasive disease”, or WNV with HIV, AIDS, “human immunodeficiency virus”, “HIV-infected”, “HIV-positive”, or “people with HIV”. The equivalent Scopus search used TITLE-ABS-KEY terms for the same WNV and HIV concepts. No language, article-type, or human filters were applied at the database-search stage.

**Figure 1:**
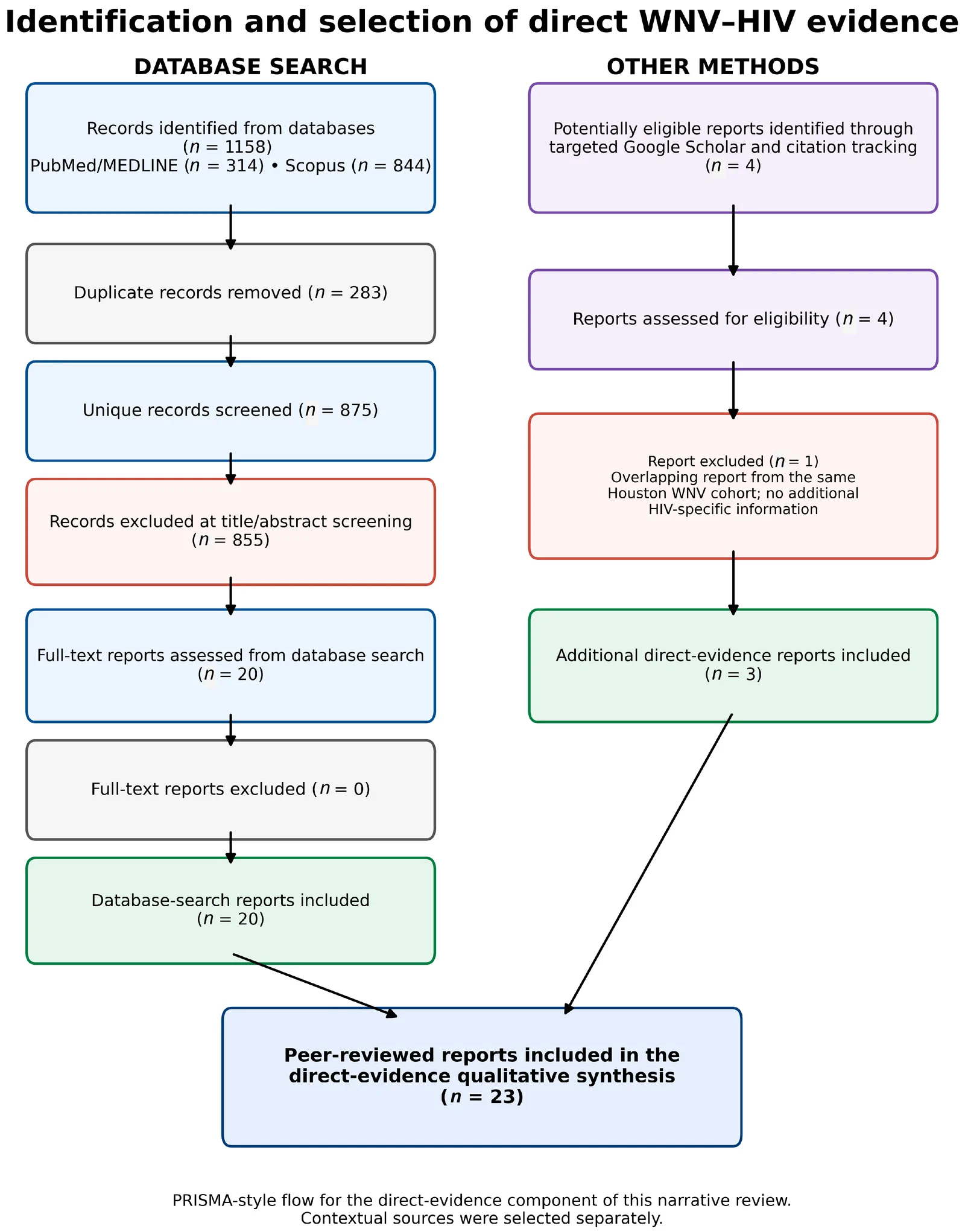
PRISMA-style flow diagram for the direct-evidence component of this narrative review. PubMed/MEDLINE and Scopus identified 20 eligible reports; targeted Google Scholar and citation tracking added three non-duplicate reports after exclusion of one overlapping report. Contextual mechanistic, diagnostic, therapeutic, preventive, and public-health sources were selected separately.

The database searches identified 1158 records (PubMed/MEDLINE, *n* = 314; Scopus, *n* = 844). Deduplication was performed using PMID, DOI, and normalized title/year, with consolidation of an earlier preprint and its subsequently published version. After removal of 283 duplicate records, 875 unique records underwent title/abstract screening. Records were retained for full-text assessment when they provided original peer-reviewed clinical or seroepidemiological data directly linking WNV infection or exposure with HIV, or when HIV was explicitly evaluated within a WNV, meningitis, or encephalitis cohort. Eight hundred fifty-five records were excluded at title/abstract screening. Twenty reports from the database search were assessed at full text and all met the direct-evidence eligibility criteria. Targeted Google Scholar searches and backward/forward citation tracking identified four additional potentially eligible peer-reviewed reports; one overlapping report from the same Houston WNV cohort did not provide additional HIV-specific information and was excluded, while three reports provided additional direct evidence. The final direct-evidence synthesis therefore comprised 23 peer-reviewed reports (Figure 1). Conference abstracts and other non-peer-reviewed reports were excluded.

Contextual sources addressing WNV epidemiology, immunopathogenesis, HIV-associated immune dysfunction, diagnostic testing, treatment, vaccination, vector control, and public-health guidance were selected separately according to relevance and were not included in the PRISMA-style flow. Google Scholar was used only as a supplementary discovery and citation-tracking tool rather than as a systematically enumerated database. Given the narrative design and marked heterogeneity of the evidence, no formal risk-of-bias assessment or quantitative synthesis was performed. Numerical statements were retained only when directly supported by the cited source.

## 3. WNV Biology, Epidemiology, and Clinical Burden

WNV is maintained through an enzootic transmission cycle in which competent avian hosts develop viremia sufficient to infect mosquitoes, primarily of the genus Culex. Humans and horses are generally incidental dead-end hosts because viremia is usually insufficient to sustain onward mosquito transmission [2,3]. This ecology makes WNV intrinsically a One Health problem: human case detection, mosquito surveillance, avian and equine surveillance, environmental monitoring, and public-health response provide complementary signals of local transmission. In Europe, ECDC surveillance integrates human data with information on outbreaks among birds and equids, supporting local risk assessment, vector-control decisions, and blood and organ safety measures [6].

The incubation period is generally 2–14 days but may be longer in immunocompromised individuals [8]. Approximately 70–80% of human infections are asymptomatic, around 20% result in an acute febrile illness, and fewer than 1% progress to WNND [7,8]. West Nile fever is typically characterized by fever, headache, fatigue, myalgia, arthralgia, gastrointestinal symptoms including nausea and vomiting, and occasionally rash or lymphadenopathy [8]. Neuroinvasive disease may present as meningitis, encephalitis, acute flaccid myelitis/paralysis, movement disorders, brainstem involvement, or mixed neurological syndromes [7].

The clinical burden of WNND extends well beyond the acute phase. Severe disease may require intensive care and ventilatory support and may result in death or persistent neurological sequelae, including weakness, cognitive impairment, fatigue, movement disorders, reduced functional independence, and prolonged rehabilitation needs [7]. Advanced age and selected comorbid or immunosuppressive conditions are consistently associated with more severe outcomes [9,10]. The expanding geographical range of WNV and the concentration of cases during seasonal peaks therefore create recurring clinical and public-health challenges, particularly for populations with reduced immune reserve.

## 4. Host Immune Control of WNV: Why Immune Status Matters

Effective control of WNV requires a coordinated sequence of innate and adaptive immune responses capable of limiting early peripheral replication, preventing systemic dissemination, restricting entry into the central nervous system, and clearing virus from infected tissues. Innate recognition of viral RNA and the subsequent induction of type I interferon responses represent an early line of defense, while complement activation, inflammatory cytokines, and chemokine-mediated recruitment of immune cells contribute to containment of infection. These responses must be appropriately timed and regulated: insufficient antiviral activity may permit sustained viremia and neuroinvasion, whereas excessive or dysregulated inflammation may contribute to neurological tissue injury. The clinical outcome of WNV infection therefore reflects not a single immune pathway, but the integration of antiviral restriction, humoral immunity, cellular effector function, and leukocyte trafficking [21,22].

Humoral immunity is particularly important during the early phase of infection. In experimental models, genetic absence of B cells and antibodies results in markedly increased viremia, wider viral dissemination, greater central nervous system viral burden, and susceptibility to lethal infection at lower viral inocula. Conversely, passive transfer of immune serum can protect susceptible animals from morbidity and mortality [23]. WNV-specific IgM contributes to early control by limiting viremia and reducing dissemination before a mature IgG response is established [24]. These observations provide a biologically plausible basis for concern in individuals with impaired antibody production, B-cell dysfunction, B-cell-depleting therapies, or delayed seroconversion. They are also diagnostically relevant because contemporary laboratory confirmation of WNV infection relies substantially on the detection of virus-specific antibodies.

Cellular immune responses become especially important for sustained control and clearance of infection from the central nervous system. In experimental models, CD4+ T-cell deficiency does not substantially alter the initial kinetics of peripheral WNV replication but results in persistent infection within the central nervous system, impaired maintenance of WNV-specific antibody responses, and reduced sustained CD8+ T-cell activity. CD4+ T cells therefore appear to provide essential help for both humoral immunity and prolonged virus-specific CD8+ T-cell function within the central nervous system [25]. CD8+ T cells, in turn, contribute to clearance of infected cells, control of viral burden in neuronal tissues, and prevention of viral persistence. Animals lacking CD8+ T cells show increased central nervous system viral loads, greater mortality, and prolonged recovery of infectious virus from neural compartments [26].

CCR5-mediated leukocyte trafficking provides an additional point of intersection between WNV immunity and HIV biology. CCR5 is widely recognized as a coreceptor for HIV entry, but it also contributes to the recruitment of immune cells to sites of WNV infection. Human genetic studies have identified homozygous CCR5 deficiency as a risk factor for symptomatic WNV infection, supporting the importance of effective immune-cell migration during host defense [27]. These findings should be interpreted cautiously: evidence derived from inherited CCR5 deficiency cannot be directly extrapolated to every form of pharmacological or biological modulation of the CCR5 pathway. Nevertheless, they illustrate how immune mechanisms relevant to HIV pathogenesis may overlap with pathways required for effective WNV control.

The translational implications of these findings require careful qualification. Most mechanistic evidence derives from experimental models rather than prospective studies in PWH. It therefore supports biological plausibility but does not demonstrate that HIV infection itself independently increases WNV susceptibility or severity. Accordingly, the individual immune phenotype—including current and nadir CD4+ T-cell count, degree of immune recovery, virological suppression, age, comorbidities, and additional immunosuppressive exposures—may be more informative than HIV status alone when interpreting potential vulnerability to WNV infection and disease.

## 5. HIV-Related Immune Dysfunction as a Potential Modifier of WNV Disease

PWH constitute an immunologically heterogeneous population. Many individuals receiving effective antiretroviral therapy achieve durable virological suppression, substantial CD4+ T-cell recovery, and long-term clinical stability. Others, however, enter care with advanced HIV disease or experience incomplete immune recovery despite virological suppression. Previous profound CD4+ T-cell depletion, persistent immune activation, immunosenescence, ageing, multimorbidity, and concomitant immunosuppressive conditions may therefore coexist to varying degrees [11,12]. Consequently, HIV status alone cannot adequately describe the immune competence of an individual potentially exposed to WNV.

Current and nadir CD4+ T-cell counts provide related but distinct information. A low current CD4+ T-cell count reflects ongoing quantitative immune impairment, whereas a low nadir CD4+ T-cell count may indicate a history of severe immune injury and incomplete immunological restoration, even after subsequent improvement during antiretroviral therapy. Persistent immune activation and immunosenescence may further affect the quality and coordination of antiviral responses despite virological suppression [11,12]. These observations do not demonstrate an HIV-specific increase in WNV risk, but they support consideration of immune recovery as a continuum rather than a binary distinction between immunocompetent and immunocompromised individuals.

HIV-associated B-cell abnormalities may also be relevant. HIV infection can affect memory B-cell compartments, antibody production, antibody quality, and responses to newly encountered antigens [13]. In the context of WNV, these abnormalities could theoretically influence both early viral control and the kinetics of seroconversion. However, there are currently no prospective studies comparing WNV-specific antibody responses between PWH and HIV-negative individuals, and the extent to which these mechanisms alter susceptibility, neuroinvasive risk, or clinical outcomes remains unknown.

The potential diagnostic implications are more immediately relevant. Laboratory confirmation of WNV infection relies primarily on the detection of WNV-specific IgM in serum or cerebrospinal fluid. Early testing may be negative before antibodies become detectable, while severe immunosuppression may delay or prevent a measurable antibody response. Public health guidance therefore recommends considering molecular testing in immunocompromised individuals, who may have prolonged viremia and delayed or absent seroconversion [28]. In PWH with advanced HIV disease, negative serology should consequently be interpreted in relation to the timing of sampling, the neurological phenotype, epidemiological exposure, and the degree of immune impairment.

**Figure 2:**
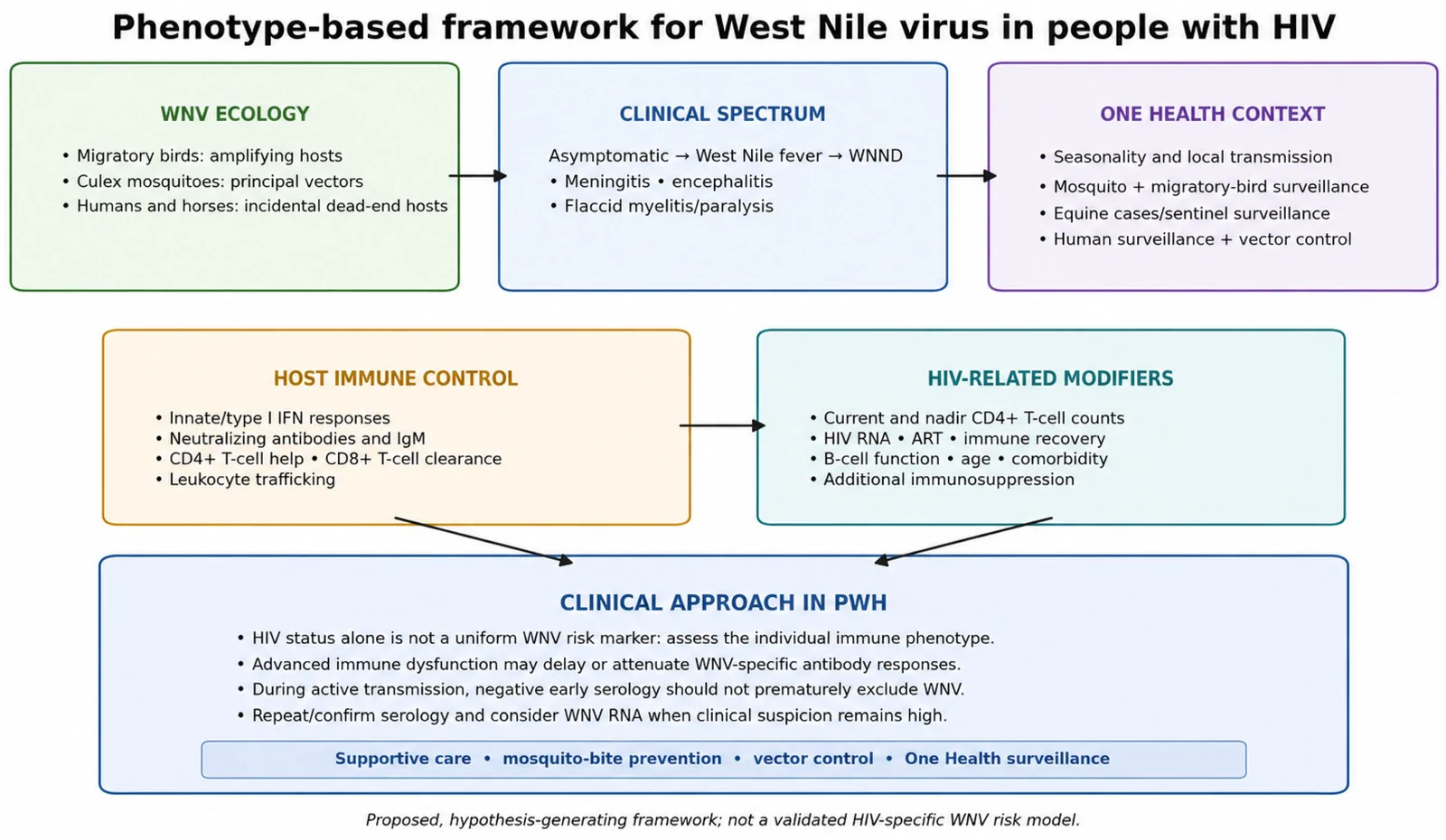
Phenotype-based framework linking WNV ecology, host immune control, HIV-related immune modifiers, and clinical implications in PWH. The framework emphasizes that HIV status alone should not be interpreted as a uniform WNV risk marker; individual immune phenotype, timing and intensity of local transmission, and diagnostic context should be considered together. In PWH with advanced immune dysfunction, delayed or attenuated WNV-specific antibody responses may justify repeat or confirmatory serology and consideration of WNV RNA when suspicion remains high. This schematic is hypothesis-generating and is not a validated HIV-specific WNV risk model.

A phenotype-based model is therefore more appropriate than categorizing all PWH as uniformly vulnerable. Available evidence does not establish a distinct WNV risk among virologically suppressed PWH with preserved immune recovery. Conversely, advanced HIV disease, low current or nadir CD4+ T-cell count, incomplete immune recovery, older age, multimorbidity, and additional immunosuppressive exposures may identify individuals in whom a lower threshold for diagnostic investigation is reasonable. This framework should be interpreted as a pragmatic approach to clinical vigilance rather than as a validated WNV risk-stratification model. This phenotype-based framework, together with its proposed clinical, diagnostic, and preventive implications, is summarized in Figure 2.

## 6. Late HIV Diagnosis and Potential WNV Vulnerability

Late HIV diagnosis is clinically relevant to the WNV–HIV intersection not because it represents an established independent risk factor for WNV disease, but because it frequently identifies individuals with low CD4+ T-cell counts, untreated HIV replication, advanced HIV disease, and limited immune reserve at the time of presentation [14,15,16,17,18,19]. These characteristics overlap with immune mechanisms involved in WNV control, including antibody production, CD4+ T-cell help, CD8+ T-cell-mediated clearance, and coordinated antiviral responses within the central nervous system [21,22,23,24,25,26]. The potential relevance of late HIV diagnosis should therefore be interpreted through the associated immune phenotype rather than through the timing of HIV diagnosis alone.

This distinction is particularly important in contemporary HIV care. Available evidence does not support considering all PWH to be uniformly vulnerable to severe WNV disease. However, individuals diagnosed with HIV at an advanced stage, particularly those with profound CD4+ T-cell depletion or untreated viremia, may warrant heightened clinical vigilance during WNV transmission seasons. In this setting, fever, meningitis, encephalitis, acute flaccid weakness, or brainstem manifestations may initially be attributed to opportunistic infections or other HIV-associated neurological conditions. WNV should nevertheless remain part of the differential diagnosis when seasonality and epidemiological exposure are compatible.

Late HIV diagnosis may also complicate laboratory confirmation. Severe immune impairment may delay or prevent detectable antibody responses, while immunocompromised individuals may experience prolonged viremia [28]. Accordingly, an initially negative WNV IgM result should not be considered conclusive when clinical and epidemiological suspicion remains high. Repeat serological testing, confirmatory neutralization, and WNV RNA testing in serum or cerebrospinal fluid should be considered according to the timing of sampling, immune status, and neurological presentation.

As late HIV diagnosis remains common across different geographical settings [17,18,19], the overlap between newly diagnosed advanced HIV disease and seasonal WNV circulation represents a plausible clinical scenario rather than an exceptional theoretical concern. The appropriate response is not to classify late HIV diagnosis as a proven WNV risk factor, but to adopt a lower threshold for diagnostic investigation and targeted prevention counselling in individuals with substantial immune impairment who live in, or have travelled through, areas of active WNV transmission.

## 7. Direct Clinical and Seroepidemiological Evidence on WNV in PWH

Direct peer-reviewed evidence on WNV infection or exposure in PWH remains limited but has expanded beyond isolated case reports. The updated evidence synthesis identified 23 eligible reports spanning the United States, Europe, and Africa, including individual clinical cases, clinicopathological observations, HIV-specific and mixed-population seroepidemiological studies, meningitis and encephalitis cohorts, and a large retrospective WNV cohort in which HIV was evaluated as a predefined comorbidity. These studies are summarized in Table 1. Their heterogeneity precludes pooled estimates of HIV-attributable risk but permits a more informative assessment of geographical exposure, clinical phenotype, testing patterns, and diagnostic challenges than was previously possible.

The geographical distribution of direct evidence is broad but uneven. Most individual clinical reports originate from the United States [29,30,31,32,33,34,35,36,40,44,45,47,49], with an additional case from Greece [39] and an imported case diagnosed in France after travel to Algeria [43]. African evidence includes population or syndrome-based studies from Ghana, Uganda, Nigeria, and Madagascar [35,38,41,46]. Comparative US meningitis and encephalitis cohorts contribute additional information on WNV detection in PWH [42,48], while the largest WNV-specific comparative cohort was also conducted in the United States [50]. The largest dedicated HIV-specific seroepidemiological cohort identified was conducted in Northern Italy [51]. This distribution reflects both WNV ecology and substantial differences in surveillance intensity, diagnostic access, and publication patterns; it should not be interpreted as a comparative map of HIV-specific WNV incidence.

HIV-specific estimates of WNV exposure remain limited and strongly dependent on assay strategy and setting. In Northern Greece, WNV IgG was detected in 3 of 91 PWH (3.3%; 95% CI, 0.7–9.3%), with no compatible symptoms reported among seropositive participants [37]. In an ART-naïve Ugandan cohort of 314 PWH evaluated for suspected meningitis, available arboviral investigations did not identify WNV: 111 CSF specimens were negative by genus-specific arboviral RT-PCR and 62 serum specimens were negative for WNV IgM [38]. In Madagascar, a Luminex-based study of 1036 PWH detected WNV-NS1 IgG in 27.1% and WNV-DIII IgG in 5.1%, with marked provincial variation; these estimates were not neutralization-confirmed and require caution because flavivirus cross-reactivity and antigen selection can materially influence apparent seroprevalence [46]. Most importantly, Tiecco et al. evaluated 2843 PWH in Northern Italy and confirmed WNV-neutralizing antibodies in 86 participants (3.0%); no WNND was reported, and seropositivity was associated much more strongly with geographical origin than with HIV-related immunological variables [51]. The Ghanaian study included substantial HIV/AIDS subgroups but did not report WNV serology separately by HIV status [35], while the Nigerian study associated HIV positivity with WNV IgM positivity but did not provide sufficiently complete HIV-stratified denominators for a reliable prevalence estimate [41]. Accordingly, the available literature does not support a single global estimate of WNV prevalence in PWH.

Comparative syndrome-based data also do not demonstrate a uniform excess of WNV among PWH, although small event numbers and testing patterns limit inference. In a US community-acquired meningitis cohort, WNV was identified in 1 of 138 PWH (0.7%) versus 14 of 411 HIV-negative participants (3.4%; *p* = 0.13) [42]. In a subsequent multicentre encephalitis cohort, WNV was identified as the etiology in one PWH versus 31 HIV-negative participants; only 15 of 40 PWH had WNV testing, and WNV IgM was positive in 1/15 tested PWH (6.7%) versus 32/120 tested HIV-negative participants (26.7%; *p* = 0.116), prompting the authors to highlight possible under-testing in PWH [48]. The largest WNV-specific comparative cohort, including 3064 adults, found HIV recorded in approximately 1% of both the WNND and West Nile fever groups (12/1127 vs. 12/1249; *p* = 0.80), and HIV was not identified as an independent predictor of WNND or mortality [50]. These data argue against treating HIV status alone as a uniform neuroinvasive-risk marker while underscoring the need for prospective studies with standardized WNV testing and detailed immune phenotyping.

The strongest clinical signal concerns diagnostic complexity in advanced immune dysfunction. Josekutty et al. reported a newly diagnosed 44-year-old man with a CD4+ T-cell count of 10 cells/mm^3^ who developed severe meningoencephalitis with bilateral thalamic and cerebral peduncle abnormalities; serum and CSF WNV serology remained negative, whereas CSF WNV RT-PCR established the diagnosis [36]. Sharma et al. subsequently described severe rhombencephalitis in a person with newly diagnosed AIDS and a CD4+ T-cell count of 44 cells/µL, again with initially negative serum and CSF antibody testing but positive molecular assays [49]. Albertson et al. reported a person with HIV/AIDS and a CD4+ T-cell count of 207 cells/µL in whom initial CSF WNV IgG/IgM ELISA was negative before a subsequent WNV IgM assay became positive [40]. These observations do not quantify the frequency of seronegative disease, but they support a lower threshold for repeat or molecular testing when profound immune dysfunction and a compatible neurological syndrome coexist.

At the same time, WNND is not restricted to advanced HIV disease. The earliest report involved a virologically suppressed woman with a CD4+ T-cell count of 351 cells/mm^3^ [29]; Pilalas et al. described WNV meningitis during lineage 2 circulation in a person with a CD4+ T-cell count of 408 cells/µL [39]; De la Selle and Henry reported WNV encephalitis in a man with long-standing, well-controlled HIV infection [43]; and Gupta and Grill described severe WNV-associated polyradiculopathy/flaccid paralysis in a person with well-controlled HIV [47]. Other reports document acute flaccid monoparesis, quadriplegia, atypical CSF findings, and overlapping autoimmune or systemic presentations across heterogeneous immune profiles [31,33,34,44,45]. A Houston WNV cohort also included a small proportion of PWH, but its immunosuppression-associated mortality estimate was a composite measure and cannot be attributed specifically to HIV [32]. This spectrum reinforces the need to integrate immune phenotype with seasonality and clinical presentation rather than using a single CD4+ T-cell threshold or HIV status alone.

Taken together, the 23 peer-reviewed reports support three cautious conclusions. First, WNV infection and WNND occur across a wide range of HIV-related immune phenotypes. Second, the best available comparative and seroepidemiological data do not demonstrate that HIV status alone confers a uniform excess risk of infection or neuroinvasive disease. Third, advanced HIV disease may be clinically relevant because profound immune dysfunction can coexist with severe neurological presentations and delayed, attenuated, or initially undetectable WNV-specific antibody responses. The evidence remains vulnerable to publication bias, incomplete immune characterization, assay heterogeneity, geographical confounding, inconsistent testing, and the scarcity of prospective HIV-specific comparator studies.

## 8. Diagnostic Approach in PWH with Suspected WNV Disease

WNV should be considered in PWH presenting during mosquito activity periods with an acute febrile illness, meningitis, encephalitis, acute flaccid weakness, rhombencephalitis, or another otherwise unexplained neurological syndrome, particularly in areas with documented WNV circulation or after travel to an endemic region. Clinical suspicion should integrate symptom onset, seasonality, geographical exposure, mosquito exposure, blood transfusion or organ transplantation history, local surveillance data, age, comorbidities, and the individual immune phenotype [7,8,28]. A pragmatic diagnostic approach to suspected WNV neuroinvasive disease in PWH is summarized in Table 2.

Detection of WNV-specific IgM antibodies in serum or cerebrospinal fluid remains the first-line laboratory approach for most individuals [28]. WNV-specific IgM generally becomes detectable within several days after symptom onset; therefore, a negative result obtained early in the clinical course may require repeat testing. Detection of WNV-specific IgM in cerebrospinal fluid supports neuroinvasive infection because IgM does not ordinarily cross the blood–brain barrier. However, IgM may persist for several months or longer, and a positive result should be interpreted in relation to symptom onset and previous flavivirus exposure.

Serological specificity represents an additional challenge. WNV antibody assays may show cross-reactivity with other flaviviruses, particularly following previous infection, vaccination, or travel to regions where dengue virus, Zika virus, Japanese encephalitis virus, yellow fever virus, tick-borne encephalitis virus, or other related flaviviruses circulate. Confirmatory plaque-reduction neutralization testing should therefore be considered when cross-reactivity is epidemiologically plausible, the presentation is unusual or severe, the outcome is fatal, or precise identification of the infecting flavivirus has clinical or public health relevance [28]. Even neutralization testing may remain inconclusive after multiple flavivirus exposures and should be interpreted alongside the clinical and epidemiological context.

In PWH with advanced HIV disease or other forms of severe immunosuppression, negative serology should not terminate the diagnostic evaluation when clinical suspicion remains high. Immunocompromised individuals may experience prolonged viremia and delayed or absent antibody responses, making WNV RNA testing in serum or cerebrospinal fluid potentially more informative than serology in selected cases [28]. Molecular testing should therefore be considered early in PWH with profound CD4+ T-cell depletion, in seronegative cases with a strongly compatible neurological syndrome, and in unusual or rapidly progressive presentations. A positive molecular result confirms acute infection, whereas a negative result does not exclude WNV disease because viral RNA may be present at low levels, transiently detectable, or confined to specific biological compartments.

The differential diagnosis of meningoencephalitis in PWH remains broad and should be guided by CD4+ T-cell count, antiretroviral therapy status, geographical exposure, clinical phenotype, cerebrospinal fluid findings, and neuroimaging. Alternative or concomitant diagnoses may include herpes simplex virus or varicella-zoster virus encephalitis, enteroviral infection, cytomegalovirus encephalitis or polyradiculomyelitis, JC virus infection, cryptococcal or tuberculous meningitis, neurosyphilis, toxoplasmosis, bacterial meningitis, Listeria monocytogenes infection, fungal infection, primary central nervous system lymphoma, autoimmune encephalitis, metabolic or drug-related encephalopathy, and cerebrovascular disease. WNV testing should therefore form part of a comprehensive diagnostic work-up rather than replace investigation for other infectious and non-infectious causes.

The practical diagnostic objective is not necessarily to establish WNV as the sole possible cause at the beginning of the evaluation, but to include it sufficiently early to avoid diagnostic delay, unnecessary or prolonged empirical treatments, and missed public health reporting. Timely diagnosis may also inform prognostic counselling, rehabilitation planning, blood and transplant safety considerations, and local arboviral surveillance.

## 9. Management, Prevention, and Integration into HIV Care

There is currently no approved or recommended specific antiviral therapy for WNV disease, and clinical management remains supportive [52]. Individuals with mild febrile illness generally require hydration, rest, and symptomatic treatment. Meningitis may require analgesia, antiemetic therapy, intravenous fluids, and monitoring. Encephalitis warrants close observation for seizures, impaired airway protection, altered consciousness, autonomic instability, and signs of increased intracranial pressure. Acute flaccid myelitis/paralysis may progress to neuromuscular respiratory failure and can require prolonged ventilatory support, multidisciplinary rehabilitation, and long-term neurological follow-up [52].

Several therapeutic approaches—including standard or high-titre immunoglobulin preparations, interferon, ribavirin, corticosteroids, and other immunomodulatory agents—have been administered empirically or evaluated in limited studies, but none has demonstrated conclusive clinical benefit or can currently be recommended as standard WNV-directed therapy [52,53]. Interest in passive antibody strategies has recently been renewed by a randomized, double-blind, placebo-controlled trial of WNV-neutralizing plasma in 34 hospitalized patients, 91% of whom had neuroinvasive disease. The primary composite outcome of death or functional deterioration at 30 days occurred in 50% of both the plasma and placebo groups; exploratory analyses suggested higher functional and cognitive scores among plasma recipients, but the small sample size and negative primary endpoint preclude routine clinical use [54].

Should potent WNV-specific monoclonal antibodies become available, PWH with advanced HIV disease or profound immune dysfunction could represent a biologically relevant population for prospective evaluation of very early therapeutic administration or, in areas with intense seasonal transmission, time-limited prophylactic strategies. This possibility is a research hypothesis rather than a clinical recommendation; dedicated studies would be required to establish efficacy, safety, optimal timing, pharmacokinetics, resistance risk, feasibility, and cost-effectiveness.

In PWH, clinical management should include assessment of HIV RNA, current and nadir CD4+ T-cell count, antiretroviral therapy status, immune recovery, comorbidities, and additional immunosuppressive exposures. Effective antiretroviral therapy should generally be continued whenever clinically feasible. In people newly diagnosed with HIV during WNV disease, no WNV-specific evidence supports a predefined delay in antiretroviral therapy initiation. Timing should therefore follow general HIV treatment principles while considering neurological stability, feasibility of enteral drug administration, potential drug–drug interactions, and the presence of concomitant central nervous system opportunistic infections for which specific recommendations regarding antiretroviral therapy timing exist [55].

Prevention remains the most actionable strategy. No WNV vaccine is currently licensed for human use, although vaccine development remains an important public-health objective [52,56,57]. In the absence of vaccination, prevention relies on mosquito-bite avoidance, household and community vector control, environmental management, epidemiological surveillance, blood donor screening, organ donor assessment, and timely reporting [52]. The One Health nature of WNV makes integration of human, mosquito, bird, and equine surveillance particularly important for anticipating local transmission and targeting vector-control measures. ECDC has emphasized the need to strengthen mosquito-control capacity as WNV spreads into new European regions [58].

Routine HIV care offers a practical setting for proportionate seasonal counselling, particularly in endemic or emerging regions. Greater diagnostic vigilance may be appropriate for older PWH and for those with advanced HIV disease, low current or nadir CD4+ T-cell counts, incomplete immune recovery, multimorbidity, or additional immunosuppressive exposures. Counselling should emphasize approved repellents, protective clothing, window and door screens, reduction in standing water, awareness of local WNV activity, and early medical evaluation for compatible febrile or neurological symptoms [5,52]. These measures are pragmatic but have not been validated as a distinct HIV-specific WNV prevention programme.

## 10. Discussion: What Can—And Cannot—Be Inferred from the Available Evidence

The updated evidence base refines the interpretation of the WNV–HIV intersection. Twenty-three peer-reviewed reports now document WNV infection or exposure in PWH across heterogeneous clinical and immunological profiles. Importantly, the evidence is no longer limited to unusual case reports: the literature includes HIV-specific seroepidemiological cohorts, comparative meningitis and encephalitis cohorts, and a large WNV cohort in which HIV was evaluated as a predefined comorbidity [42,48,50,51]. Together, these data argue against considering HIV status alone as a uniform determinant of WNV susceptibility, neuroinvasive disease, or outcome, while also revealing important limitations related to low event counts and incomplete WNV testing in some PWH cohorts.

The most clinically relevant signal remains diagnostic complexity in advanced HIV disease. Seronegative neuroinvasive WNV infection confirmed by molecular testing has been reported in individuals with profound CD4+ T-cell depletion [36,49], while another immunocompromised case had initially negative WNV antibody testing before subsequent IgM detection [40]. These observations are biologically compatible with delayed or attenuated humoral responses, but they do not establish how frequently this occurs or define an evidence-based CD4+ T-cell threshold for molecular testing. The practical implication is therefore conditional: in a compatible neurological syndrome during active WNV transmission, profound immune dysfunction should lower the threshold for repeat serology, neutralization, and WNV RNA testing rather than automatically changing the initial diagnostic algorithm.

Mechanistic evidence provides biological plausibility for a role of the individual immune phenotype. Effective WNV control requires coordinated innate antiviral responses, neutralizing antibodies, CD4+ T-cell help, CD8+ T-cell-mediated viral clearance, and appropriate leukocyte trafficking [21,22,23,24,25,26,27]. Advanced HIV disease may disrupt several of these pathways simultaneously, while incomplete immune recovery, ageing, multimorbidity, and additional immunosuppressive exposures may further reduce host resilience [11,12,13]. However, most mechanistic evidence derives from experimental models rather than prospective HIV-specific studies and should therefore be interpreted as contextual support rather than direct proof of excess risk.

Recent seroepidemiological data also highlight the importance of geography and assay methodology. In the Northern Italian cohort, neutralization-confirmed WNV seroprevalence was 3.0%, no WNND was observed, and geographical origin was a substantially stronger correlate of seropositivity than HIV-related immunological measures [51]. By contrast, WNV-reactive antibody prevalence in Madagascar varied markedly according to antigen and province, illustrating why cross-study prevalence comparisons require caution [46]. Negative or low-frequency WNV findings in HIV-specific meningitis cohorts from Uganda and the United States further emphasize that observed burden depends on setting, syndrome selection, and testing strategy [38,42]. These findings reinforce a One Health and exposure-based interpretation: local viral circulation, vector ecology, travel, and prior flavivirus exposure may be at least as important as HIV-related variables when estimating the probability of WNV infection.

Comparative clinical datasets likewise do not identify HIV status alone as a consistent marker of WNV neuroinvasive disease [42,48,50]. The largest WNV-specific cohort did not identify HIV as an independent risk factor for WNND or mortality [50]. However, the encephalitis cohort demonstrated that WNV testing was performed in fewer than half of PWH, illustrating how ascertainment can distort apparent between-group differences [48]. These studies do not exclude clinically important vulnerability in selected PWH, because available datasets incompletely capture nadir CD4+ T-cell counts, immune recovery, duration of advanced HIV disease, or other immunological dimensions. They do, however, argue against broad statements that PWH as a whole are uniformly at increased risk of neuroinvasive disease. A phenotype-based approach is therefore more defensible than a status-based approach.

Despite these limitations, several practical implications emerge. During documented WNV circulation, clinicians should include WNV early in the differential diagnosis of compatible febrile or neurological syndromes. HIV RNA, current and nadir CD4+ T-cell counts, ART status, comorbidities, and additional immunosuppressive exposures should be documented as part of clinical phenotyping. Serum and CSF WNV-specific IgM remain first-line diagnostic tests, but an early negative result should not prematurely terminate evaluation in a profoundly immunocompromised person when clinical and epidemiological suspicion remains high. Repeat serology, confirmatory neutralization, and WNV RNA testing should be considered according to timing and phenotype [28].

Prevention should be integrated with local surveillance rather than framed as an HIV-specific intervention. Brief seasonal counselling on mosquito-bite prevention can be incorporated into routine HIV care, while vector control and human, mosquito, avian, and equine surveillance require coordinated public-health action [6,52,58]. The absence of an approved human vaccine or established antiviral treatment increases the importance of these measures and supports continued development of vaccines and antibody-based countermeasures [54,56,57].

Several limitations of the evidence and of this review should be acknowledged. The direct literature remains dominated by case reports and small observational studies, creating publication bias toward severe or unusual neurological phenotypes. HIV-related variables are inconsistently reported, diagnostic methods differ substantially, and seroepidemiological estimates are influenced by assay specificity and cross-reactivity. Comparative meningitis and encephalitis cohorts contain few WNV events and may be affected by incomplete or non-standardized testing [42,48], while the largest WNV-specific comparative cohort was retrospective and not designed specifically around HIV immune phenotypes [50]. In addition, this work remains a narrative review: the PRISMA-style flow was used to document selection of the direct WNV–HIV evidence component, but no formal risk-of-bias tool or quantitative meta-analysis was applied. These limitations preclude reliable estimates of HIV-attributable incidence, relative risk, diagnostic accuracy, or prognosis.

Overall, current evidence supports a phenotype-based interpretation of possible WNV vulnerability in PWH. HIV status alone provides insufficient clinical information. The combination of current and nadir CD4+ T-cell counts, virological control, degree of immune recovery, age, comorbidities, concomitant immunosuppression, and geographical/seasonal exposure provides a more clinically meaningful framework for assessment. This approach remains hypothesis-generating and should not be regarded as a validated risk model, but it better reflects the available evidence than a generalized assumption of HIV-associated WNV risk.

## 11. Future Directions and Research Priorities

Future research should build on the newly expanded evidence base by moving from isolated case descriptions toward coordinated prospective investigation. Priorities include standardized HIV-specific serosurveillance using confirmatory neutralization where feasible, prospective characterization of diagnostic antibody and RNA kinetics across immune phenotypes, longitudinal neurological outcomes, and evaluation of emerging vaccines and antibody-based countermeasures. These gaps and proposed approaches are summarized in Table 3.

Prospective multicentre registries remain an immediately feasible strategy. A minimum dataset should include geographical and seasonal exposure, age, sex, current and nadir CD4+ T-cell counts, CD4/CD8 ratio, HIV RNA, ART status, comorbidities, additional immunosuppressive therapies, clinical presentation, CSF findings, neuroimaging, timing and results of serological and molecular testing, acute management, and neurological follow-up. Harmonized reporting would allow future studies to separate HIV-related immune effects from age, comorbidity, geography, and healthcare ascertainment.

Diagnostic studies should specifically examine whether advanced HIV disease, incomplete immune recovery, or profound CD4+ T-cell depletion alters the kinetics or sensitivity of WNV-specific antibody responses. The molecularly confirmed seronegative cases and the initially antibody-negative case reported to date [36,40,49] justify prospective evaluation of paired serum and CSF testing, repeat serology, neutralization assays, and WNV RNA according to timing of illness and immune phenotype. Comparative encephalitis data also show that incomplete WNV testing in PWH can complicate interpretation of apparent between-group differences [48]. Such studies could determine whether selected PWH benefit from earlier molecular testing without imposing an unvalidated CD4+ T-cell threshold.

Late HIV diagnosis warrants dedicated investigation. Future registries should apply standardized definitions of late presentation and record the timing of HIV diagnosis in relation to WNV disease [14,15]. This would help clarify whether late diagnosis simply identifies profound underlying immune dysfunction or is associated with distinctive diagnostic patterns, neurological phenotypes, or outcomes. The objective should not be to classify late HIV diagnosis prematurely as an independent WNV risk factor, but to determine whether it improves clinical phenotyping and diagnostic decision-making.

Finally, implementation and countermeasure research should connect HIV care with One Health surveillance. Brief seasonal counselling, electronic prompts linked to local WNV activity, and standardized diagnostic pathways could be evaluated for feasibility and clinical impact. Prospective studies should also determine whether future WNV-specific monoclonal antibodies or other passive-immunity strategies have a role in very early treatment or time-limited prophylaxis for selected profoundly immunocompromised populations. Collaboration among HIV specialists, neurologists, microbiologists, blood/transplant services, public-health authorities, entomologists, and animal-health surveillance programmes will be essential.

## 12. Conclusions

This review identifies a clinically relevant but still incompletely characterized intersection between WNV infection and HIV. Current evidence, including HIV-specific seroepidemiological and comparative cohort data, does not support HIV status alone as a uniform determinant of WNV infection, neuroinvasive disease, or mortality [42,48,50,51]. The most actionable signal concerns selected PWH with advanced immune dysfunction, in whom delayed or initially undetectable WNV-specific antibody responses have been reported [36,40,49]. During periods of active WNV transmission, clinicians should therefore: (i) include WNV early in the differential diagnosis of compatible febrile or neurological syndromes; (ii) document current and nadir CD4+ T-cell counts, HIV RNA, ART status, comorbidities, and additional immunosuppression; (iii) avoid excluding WNV solely on the basis of negative early serology when profound immunosuppression and strong clinical suspicion coexist; (iv) consider repeat serology, confirmatory neutralization, and WNV RNA testing according to timing and phenotype; and (v) reinforce seasonal mosquito-bite prevention and appropriate public-health reporting. Comparative cohorts also indicate that standardized WNV testing is essential before apparent differences by HIV status are interpreted biologically. In the absence of an approved human vaccine or proven WNV-specific therapy, prevention, supportive care, surveillance, and timely diagnosis remain central. Prospective multicentre studies should now quantify HIV-specific burden, define antibody and RNA kinetics across immune phenotypes, and evaluate emerging antibody-based countermeasures.

## Figures and Tables

**Table 1:** Direct peer-reviewed clinical and seroepidemiological evidence on West Nile virus infection or exposure in people with HIV.

Study	Country/Setting, Design, and HIV-Related Characteristics	WNV Phenotype/Exposure and Diagnostic Evidence	Outcome/Key Contribution
Szilak and Minamoto, 2000 [29]	United States; case report/letter HIV: 38-year-old woman with HIV; CD4+ T-cell count 351 cells/mm^3^; HIV RNA < 50 copies/mL; receiving ART	Febrile meningitis/encephalitis-like illness; CSF WNV-specific IgM detected	Demonstrates WNND in a virologically suppressed person with a moderately preserved CD4+ T-cell count.
Guarner et al., 2004 [30]	United States; clinicopathological series of 23 fatal WNV encephalomyelitis cases HIV: Two cases had AIDS; individual CD4+ T-cell counts and HIV-related clinical details were not reported	Fatal WNV encephalomyelitis confirmed by serology and/or CNS immunohistochemistry	AIDS was represented among fatal WNND cases, but insufficient case-level data preclude HIV-specific risk inference.
Unzek et al., 2006 [31]	United States; case report HIV: 45-year-old man with advanced HIV; CD4+ T-cell count 128 cells/mm^3^ as summarized in a subsequent report	Meningoencephalitis/meningitis-like syndrome with lymphocytic pleocytosis and progressive hypoglycorrhachia; serological diagnosis	Highlights an atypical CSF profile in advanced HIV disease that may broaden the differential diagnosis.
Murray et al., 2006 [32]	Houston, United States; nested case–control/retrospective chart review of 172 hospitalized laboratory-confirmed WNV cases HIV: HIV infection was present in approximately 2% of the cohort; case-specific CD4+ T-cell and HIV RNA data were not reported	113 encephalitis, 47 meningitis, and 12 West Nile fever cases; 17 deaths. A composite immunosuppression variable that included HIV was associated with death after age adjustment, but the effect was not HIV-specific	Confirms that PWH were represented in a large hospitalized WNV cohort, but the study does not permit HIV-specific attribution of neuroinvasive risk or mortality.
Torno et al., 2007 [33]	United States; case report and literature review HIV: 47-year-old man; ART-naïve; CD4+ T-cell count 324 cells/µL; HIV RNA 25,012 copies/mL	WNV infection presenting with acute flaccid monoparesis of the right upper extremity; WNV IgM positive	Documents a poliomyelitis-like phenotype in HIV; neurological improvement/partial recovery was reported.
Jamison et al., 2007 [34]	United States; case report HIV: 41-year-old man with HIV; CD4+ T-cell count 93 cells/mm^3^	Fever with acute quadriplegia/quadriparesis and hearing impairment; WNV serology positive	Illustrates severe motor involvement in advanced immunosuppression and the need for prolonged rehabilitation.
Wang et al., 2009 [35]	Ghana; molecular and seroepidemiological study, *n* = 1324 HIV: Adult groups included 203 symptomatic PWH and 130 people with AIDS	No WNV RNA detected; adult WNV IgG prevalence 27.9% overall, not stratified by HIV status	Provides endemic-setting evidence but does not permit an HIV-specific prevalence estimate.
Josekutty et al., 2013 [36]	United States; case report HIV: 44-year-old man newly diagnosed with HIV; CD4+ T-cell count 10 cells/mm^3^	Severe meningoencephalitis with bilateral thalamic/cerebral peduncle involvement; serum and CSF serology negative; CSF WNV RT-PCR positive	Key evidence of seronegative WNND in advanced HIV disease and of the diagnostic value of molecular testing.
Pilalas et al., 2015 [37]	Northern Greece; prospective seroprevalence study HIV: 91 consecutive PWH receiving HIV care	WNV IgG detected in 3/91 (3.3%; 95% CI, 0.7–9.3%); no compatible illness reported among seropositive participants	Provides an HIV-specific European exposure estimate in an endemic/outbreak setting.
Rajasingham et al., 2015 [38]	Uganda; prospective cohort of 314 ART-naïve PWH with suspected meningitis HIV: HIV-specific cohort; extensive evaluation for opportunistic and arboviral causes	Among samples with sufficient material, 111 CSF specimens were negative by genus-specific arboviral RT-PCR and 62 serum specimens were negative for WNV IgM	Provides substantial negative HIV-specific evidence from East Africa and illustrates the rarity of identified WNV in an intensively investigated meningitis cohort.
Pilalas et al., 2017 [39]	Greece; case report HIV: 35-year-old man; ART-naïve; AIDS-related Kaposi sarcoma; CD4+ T-cell count 408 cells/µL; HIV RNA 17,806 copies/mL	Viral meningitis during regional WNV lineage 2 circulation; subsequent WNV IgM/IgG and neutralization confirmation	Supports outbreak-season diagnostic vigilance; clinical recovery was reported.
Albertson et al., 2018 [40]	United States; case report/clinical problem-solving report HIV: 41-year-old man with HIV/AIDS and previous PML; CD4+ T-cell count 207 cells/µL	Rapidly progressive flaccid paralysis; initial CSF WNV IgG/IgM ELISA negative; subsequent WNV IgM positive by microsphere immunoassay	Shows that an initially negative antibody assay may be misleading in an immunocompromised person; substantial residual motor disability persisted.
Kolawole et al., 2018 [41]	Ilorin, Nigeria; cross-sectional study of 200 febrile participants HIV: HIV status evaluated as a clinical risk factor	15/200 participants (7.5%) were WNV-IgM positive; HIV positivity was reported as associated with WNV positivity (*p* = 0.003)	Suggests WNV–HIV overlap in a febrile African population, but incomplete HIV-stratified denominators preclude a reliable HIV-specific prevalence estimate.
Vigil et al., 2018 [42]	New Orleans and Houston, United States; retrospective community-acquired meningitis cohort, *n* = 549 HIV: 138 PWH and 411 HIV-negative participants; median CD4+ T-cell count in the HIV group 89 cells/mm^3^ (case-specific CD4 not reported)	WNV was identified in 1/138 PWH (0.7%) versus 14/411 HIV-negative participants (3.4%); *p* = 0.13	Comparative meningitis data did not show excess WNV frequency in PWH, although the small number of WNV events limits inference.
De la Selle and Henry, 2020 [43]	France; imported infection after travel to Algeria; case report HIV: 57-year-old man with long-standing, well-controlled HIV infection; not described as severely immunocompromised	WNV encephalitis with behavioral disturbance, tremor, parkinsonism, ataxia, and lymphocytic meningitis; serological confirmation	Demonstrates neuroinvasive WNV disease in a person with well-controlled HIV, arguing against restriction of WNND to advanced HIV disease.
Momah and Mbride, 2020 [44]	United States; case report HIV: 25-year-old woman with concomitant new HIV diagnosis; detailed immune status not reported	Concurrent HIV and WNV diagnoses after a prolonged febrile multisystem illness with headache, arthralgia, nausea, and vomiting	Highlights clinical overlap between acute HIV presentation and WNV infection.
Arcilla et al., 2023 [45]	United States; clinical letter/case observation HIV: HIV infection; detailed case-level immune data limited	WNV encephalitis associated with amphiphysin antibodies and rapid neurological deterioration	Raises the possibility of immune-mediated neurological overlap; causal interpretation remains uncertain.
Rakotomalala et al., 2023 [46]	Madagascar; multicentre serological study HIV: 1036 PWH from five provinces; approximately 70% receiving ART	WNV-NS1 IgG 27.1% (95% CI, 24.4–29.8%); WNV-DIII IgG 5.1% (95% CI, 3.8–6.5%) using a Luminex-based assay	Shows geographically heterogeneous WNV-reactive antibodies in PWH; estimates are assay-dependent and were not neutralization-confirmed.
Gupta and Grill, 2023 [47]	Arizona, United States; case report HIV: 60-year-old man with well-controlled HIV	Abrupt lower-extremity weakness progressing to severe paraparesis; cauda equina ventral-root enhancement; laboratory-confirmed WNND with flaccid paralysis	Broadens the reported phenotype to severe neuromuscular WNND despite well-controlled HIV infection.
Reimer-McAtee et al., 2023 [48]	Houston, United States; multicentre retrospective encephalitis cohort, *n* = 260 HIV: 40 PWH and 220 HIV-negative participants; WNV-specific CD4+ T-cell data were not reported	WNV was identified as the etiology in 1 PWH versus 31 HIV-negative participants. WNV IgM testing was positive in 1/15 tested PWH (6.7%) versus 32/120 tested HIV-negative participants (26.7%); *p* = 0.116	The authors highlighted possible under-testing because only 15/40 PWH were evaluated for WNV; lower observed detection therefore should not be interpreted as lower biological risk.
Sharma et al., 2024 [49]	United States; case report HIV: 26-year-old man newly diagnosed with AIDS; CD4+ T-cell count 44 cells/µL; HIV RNA 19,000 copies/mL	Severe rhombencephalitis, seizures, and coma; initial serum/CSF WNV IgM/IgG negative; serum and CSF qualitative PCR positive	Reinforces the possibility of seronegative, diagnostically delayed, severe WNND in advanced HIV disease.
Judson and Dowdy, 2025 [50]	United States; retrospective multicentre EHR cohort of 3064 WNV cases HIV: HIV included as a predefined comorbidity; HIV prevalence was similar in WNND and WNF groups (12/1127 vs. 12/1249; approximately 1% vs. 1%; *p* = 0.80)	WNV cases classified as WNF or WNND; HIV was not identified as an independent predictor of WNND or mortality	Strongest comparative WNV-specific evidence identified; supports avoiding HIV status alone as a uniform WNND risk marker.
Tiecco et al., 2026 [51]	Brescia, Northern Italy; retrospective sero-repository cohort HIV: 2843 PWH; mean current CD4+ T-cell count 801 cells/mm^3^; 94.2% virologically suppressed and on stable ART	Neutralization-confirmed WNV antibodies in 86/2843 (3.0%); no WNND reported; no meaningful association with current/nadir CD4-related measures	Largest neutralization-confirmed HIV-specific seroepidemiological study identified; geographic origin was a stronger correlate of seropositivity than HIV-related immune variables.

Abbreviations: AIDS, acquired immunodeficiency syndrome; ART, antiretroviral therapy; CD4, cluster of differentiation 4; CI, confidence interval; CNS, central nervous system; CSF, cerebrospinal fluid; EHR, electronic health record; HIV, human immunodeficiency virus; IgG, immunoglobulin G; IgM, immunoglobulin M; PML, progressive multifocal leukoencephalopathy; PWH, people with HIV; RNA, ribonucleic acid; RT-PCR, reverse-transcription polymerase chain reaction; WNF, West Nile fever; WNND, West Nile neuroinvasive disease; WNV, West Nile virus. Seroprevalence estimates should not be directly compared across studies without considering assay platform, neutralization confirmation, population selection, and geographical exposure.

**Table 2:** Pragmatic diagnostic approach to suspected West Nile virus infection or neuroinvasive disease in people with HIV.

Clinical Scenario	Initial Assessment and Testing	When to Escalate	Interpretive Considerations
Acute fever, headache, myalgia, rash, or gastrointestinal symptoms during WNV transmission season	Assess local WNV activity, mosquito exposure, travel, transfusion or transplantation history, age, comorbidities, HIV RNA, and current and nadir CD4+ T-cell count. Consider serum WNV-specific IgM when the epidemiological context is compatible.	Escalate the diagnostic evaluation if neurological symptoms develop, symptoms are severe or prolonged, or advanced HIV disease or additional immunosuppression is present.	Non-neuroinvasive WNV disease is clinically nonspecific and overlaps with other viral and systemic illnesses. A negative early IgM result does not exclude infection.
Meningitis, encephalitis, or rhombencephalitis	Perform CSF examination and serum and CSF WNV-specific IgM testing. Include HSV, VZV, enterovirus, bacterial cultures, cryptococcal testing, and other investigations according to immune status and epidemiology. Obtain neuroimaging when clinically indicated.	Request neutralizing antibody testing, including PRNT, when flavivirus cross-reactivity is plausible or the presentation is unusual, severe, or fatal. Consider WNV RT-PCR in serum and CSF when severe immunosuppression or persistently negative serology complicates diagnosis.	CSF WNV-specific IgM supports neuroinvasive infection, but early sampling, antibody persistence, cross-reactivity, and impaired antibody responses may complicate interpretation.
Advanced HIV disease or other severe immunosuppression with a compatible clinical syndrome	Do not rely on serology alone. Obtain serum and CSF WNV-specific IgM and consider early WNV RNA testing in serum and CSF where available. Document HIV RNA, current and nadir CD4+ T-cell count, ART status, and additional immunosuppressive exposures.	Repeat serological testing when the initial sample was collected early or results remain discordant. Consult public health or reference laboratories regarding molecular and neutralization testing.	Delayed or absent antibody responses may produce false-negative IgM results, while viremia may be prolonged. A negative RT-PCR result does not exclude WNV disease. No CD4+ T-cell count threshold has been validated specifically for selecting molecular testing.
Acute flaccid weakness, asymmetric paralysis, respiratory weakness, or rhombencephalitis	Include WNV early in the differential diagnosis during transmission seasons. Perform MRI of the brain and/or spine and neurophysiological testing as clinically indicated, including evaluation for anterior horn cell involvement.	Consider ICU monitoring when respiratory or bulbar dysfunction is present. Add WNV RT-PCR when serology is negative but clinical and epidemiological suspicion remains high.	Differential diagnoses include Guillain-Barre syndrome, enteroviral infection, CMV polyradiculomyelitis, spinal cord disease, metabolic abnormalities, and other causes of acute neuromuscular weakness.
Previous flavivirus infection or vaccination, or travel to regions with circulation of other flaviviruses	Interpret WNV IgM and IgG results in the context of previous infection, vaccination, geographical exposure, and local arboviral circulation.	Request PRNT or another reference-laboratory neutralization assay when precise identification of the infecting flavivirus has clinical or public health relevance.	Serological cross-reactivity may occur after dengue, Zika, yellow fever, Japanese encephalitis, tick-borne encephalitis, or other flavivirus exposures. IgG positivity alone generally indicates previous exposure rather than acute WNV disease.

Abbreviations: ART, antiretroviral therapy; CD4, cluster of differentiation 4; CMV, cytomegalovirus; CSF, cerebrospinal fluid; HIV, human immunodeficiency virus; HSV, herpes simplex virus; ICU, intensive care unit; IgG, immunoglobulin G; IgM, immunoglobulin M; MRI, magnetic resonance imaging; PRNT, plaque-reduction neutralization test; RNA, ribonucleic acid; RT-PCR, reverse-transcription polymerase chain reaction; VZV, varicella-zoster virus; WNV, West Nile virus.

**Table 3:** Research gaps and proposed priorities for West Nile virus infection in people with HIV.

Knowledge Gap	Why It Matters	Proposed Approach
Incidence and clinical burden of WNV infection in PWH	Recent HIV-specific serosurveillance and comparative cohort data improve the evidence base, but incidence, geographical variation, and risk relative to people without HIV remain incompletely defined.	Link established cohorts of PWH with seasonal arboviral surveillance; use standardized, preferably neutralization-confirmed serology; conduct prospective multicentre studies with suitable comparison groups.
Role of the individual HIV-related immune phenotype	Potential vulnerability may depend more on immune status and recovery than on HIV status alone.	Standardize collection of current and nadir CD4+ T-cell count, CD4/CD8 ratio, HIV RNA, ART status and duration, degree of immune recovery, age, comorbidities, and additional immunosuppressive exposures. Evaluate their association with infection, neuroinvasive disease, diagnostic phenotype, and clinical outcome.
Diagnostic performance of serology and molecular testing	Delayed or absent antibody responses and seronegative neuroinvasive disease have been reported in advanced HIV disease, but the frequency and predictors of these presentations are unknown.	Prospectively evaluate paired serum and CSF WNV-specific IgM, neutralizing antibodies, and WNV RNA according to immune phenotype, timing of illness, clinical syndrome, and specimen type. Define when repeat serology or early molecular testing provides additional diagnostic value.
Long-term neurological and functional outcomes	WNND may result in persistent motor, cognitive, and functional impairment, but HIV-specific recovery trajectories have not been characterized.	Collect standardized longitudinal data on neurological recovery, cognition, functional status, quality of life, rehabilitation requirements, return to work, and mortality. Compare outcomes according to immune status and clinical phenotype.
Clinical relevance of late HIV diagnosis and advanced HIV disease	Late HIV diagnosis may identify individuals presenting with profound immune dysfunction, potentially atypical serological responses, and complex neurological syndromes, but it has not been established as an independent WNV risk factor.	Apply standardized definitions of late HIV presentation and record timing of HIV diagnosis, baseline and nadir CD4+ T-cell count, HIV RNA, AIDS-defining conditions, ART initiation, and subsequent immune recovery in WNV case-reporting templates and multicentre registries.
Effectiveness of seasonal prevention, One Health surveillance, and counselling	Routine HIV care may support prevention messages, but effectiveness is unknown and WNV risk is strongly shaped by local vector, bird/equine, and human transmission patterns.	Evaluate brief seasonal counselling and electronic prompts linked to local surveillance; integrate HIV services with vector-control, entomological, avian/equine, blood-safety, and public-health systems.
Geographical and temporal heterogeneity	WNV circulation varies substantially by region and season, potentially limiting the generalizability of findings from individual centres or outbreaks.	Develop international collaborations linking HIV cohorts, infectious disease units, neurological services, public health laboratories, and vector-surveillance programmes. Use harmonized case definitions and minimum reporting datasets across different epidemiological settings.
Antibody-based therapeutics and vaccination	No human WNV vaccine is licensed, and no specific therapy is established. A small randomized trial of neutralizing plasma did not improve its primary endpoint, while exploratory functional/cognitive signals and future monoclonal antibodies justify further study.	Evaluate vaccines and WNV-specific monoclonal antibodies in appropriately designed trials. If potent antibodies become available, assess very early treatment and carefully selected seasonal prophylaxis strategies in profoundly immunocompromised populations.

Abbreviations: AIDS, acquired immunodeficiency syndrome; ART, antiretroviral therapy; CD4, cluster of differentiation 4; CD8, cluster of differentiation 8; CSF, cerebrospinal fluid; HIV, human immunodeficiency virus; IgM, immunoglobulin M; PWH, people with HIV; RNA, ribonucleic acid; WNND, West Nile neuroinvasive disease; WNV, West Nile virus.

## Data Availability

No new data were created or analyzed in this study. Data sharing is not applicable to this article.
